# Gut microbiome alterations in ICU patients with enteral nutrition-related diarrhea

**DOI:** 10.3389/fmicb.2022.1051687

**Published:** 2022-11-22

**Authors:** Weiwei Ni, Xinwei Jiao, Huihuang Zou, Mengjuan Jing, Ming Xia, Shichao Zhu, Liming Li

**Affiliations:** ^1^Department of Intensive Care Unit, Henan Provincial People’s Hospital, People’s Hospital of Zhengzhou University, Zhengzhou, China; ^2^Department of Pathophysiology, Jinan University Medical School, Guangzhou, China

**Keywords:** gut microbiome, enteral nutrition, diarrhea, ICU, 16S rRNA sequencing

## Abstract

Enteral Nutrition-related Diarrhea (END) is an extremely common complication in Intensive Care Unit (ICU) patients. However, it is currently unclear whether the patient’s gut microbiota is disturbed. Our study aimed to explore the characteristics of gut microbiota changes in END patients. We divided ICU patients into no-END group (*n* = 7) and END group (*n* = 7) according to whether they had END, then stool samples were collected separately. The V3-V4 region of stool bacterial 16S rRNA gene was amplified by PCR and sequenced on an Illumina MiSeq PE300 platform. Microbiome data obtained by quality control were analyzed, including microbial community composition, diversity and gene function prediction.The results showed that the dominant gut microbiota in ICU patients who were given total enteral nutrition were Firmicutes, Proteobacteria, Bacteroidetes, Actinobacteria, and Verrucomicrobia. Bacterial richness and diversity in END patients were all significantly lower than those in no-END patients. In addition, END caused significant changes in bacterial composition. LEfSe found 34 biomarkers represented by Bacteroidetes and Subdoligranulum in the no-END group as well as 11 biomarkers represented by Enterococcus and Klebsiella in the END group. Finally, through PICRUST function prediction, we found that diarrhea led to abnormal changes in numerous KEGG pathways mainly related to immunity and metabolism. In short, ICU patients with END have severe gut dysbiosis, and our study provides a reliable experimental basis for the patient’s microbiota therapy.

## Introduction

Intensive care unit (ICU) patients are generally in critical diseases, often accompanied by disorders of consciousness, dysphagia, digestive dysfunction and other states, and are prone to malnutrition (Singer, 2019; Theilla et al., 2021). Enteral nutrition (EN) is a form of oral or tube feeding that provides food and other nutrients to the gastrointestinal tract of people who cannot eat normally (Wischmeyer, 2021). Early nutritional support treatment helps to correct electrolyte and various nutritional metabolic disorders in patients and also effectively improves the body’s resistance, shortens the patient’s stay in the ICU and reduces the mortality rate (Singer et al., 2019; De Lazzaro et al., 2022). The 2009 Nutrition Day Global Hospital Survey reports that approximately 10% of hospitalized patients in Europe and Japan receive enteral nutrition, with one-third using it as part of their energy supplement and another two-thirds using it as their sole energy source (Schneider, 2010). EN is the preferred route of feeding, especially when the oral route can no longer meet the needs of patients (Lochs et al., 2006). The European Society of Clinical Nutrition and Metabolism’s 2018 nutrition guidelines for critically ill patients strongly recommend early enteral nutrition within 48 h of admission to the hospital for critically ill patients who are unable to eat autonomously (Singer et al., 2019). However, diarrhea is the most common complication of enteral nutrition, with an incidence rate as high as 68% (Zhao et al., 2017; Xie et al., 2021). Surprisingly, the incidence of diarrhea in ICU patients treated with EN is as high as 2–95% (Wiesen et al., 2006; Whelan, 2007). The consequences of enteral nutrition-related diarrhea (END) range from ICU patient discomfort to life-threatening acidosis, increased morbidity and mortality, pressure ulcers and the forced interruption of EN feeding, as well as higher care costs for hospital staff (Adam and Batson, 1997). Unfortunately, when END occurs, some ICU patients experience long-lasting comas due to their critical condition; thus, EN cannot be easily stopped as it is the only source of nutrition. Therefore, it is extremely important to explore the mechanism of END in ICU patients.

The gut microbiome is considered a new functional organ, as it regulates many physiological functions of the host, such as training of immunity, digesting food, regulating gut endocrine function and nerve signals, modifying drug action and metabolism, eliminating toxins and producing numerous compounds that influence the host (Fan and Pedersen, 2021). Undoubtedly, microbiota, nutrients and host cells interact extensively in the gut, and this process is critical for gut homeostasis and host health. Therefore, any dysbiosis can have a negative impact on health, and many diseases are associated with damage to the gut microbiota (Butel, 2014). Studies have found that the concentration and proportion of Clostridium and *Bacteroides* are significantly higher in patients with diarrhea (Whelan et al., 2009). Long-term total EN patients have a serious imbalance of the bacterial community; for example, the number of fecal anaerobic bacteria decreases, the number of aerobic bacteria increases, and the cultured strains decrease significantly (Schneider et al., 2000). Other studies have found that the detection rate of *Escherichia coli* in children under 5 years old with acute diarrhea is 7.9% (Zhou et al., 2018). Interestingly, gastrointestinal disorders, such as autism spectrum disorders and diarrhea in children, are closely related to gut microbiota disorders (Krajmalnik-Brown et al., 2015). Fecal bacteria transplantation can improve the gastrointestinal tract and symptoms of autism (Kang et al., 2017). Although, there have been numerous studies on the relationship between diarrhea and the gut microbiota, the comprehensive and detailed changes in gut microbiota composition and gene function during END in ICU patients are still unknown. Moreover, most previous studies examined the relationship between gut microbiota and diarrhea using traditional identification and culture techniques that are no longer considered optimal for describing the microbiota (Hooda et al., 2012; Braun et al., 2017; Pena-Gonzalez et al., 2019). New molecular biology techniques, especially next-generation sequencing (NGS) technology, such as 16S rRNA gene sequencing and metagenomics, because it can simultaneously detect the dominant species, rare species and some unknown species in the sample quickly and high-throughput, without considering whether they are known and culturable. In addition, microbial community composition, diversity, evolutionary relationships and even gene function can be more accurately studied, and the discovery and classification of new bacterial species can be facilitated. Thus, the emergence of NGS has largely replaced the old methods (Okonkwo et al., 2020).

In this study, 16S rRNA gene sequencing was used to detect stool from ICU patients with and without diarrhea receiving total enteral nutrition to compare the gut microbiota changes. Our data show that END patients have distinct gut microbial signatures and significant changes in gene function. These results are important for understanding the roles of these bacteria in the occurrence of END.

## Materials and methods

### Study subjects and sample collection

**Figure 1:**
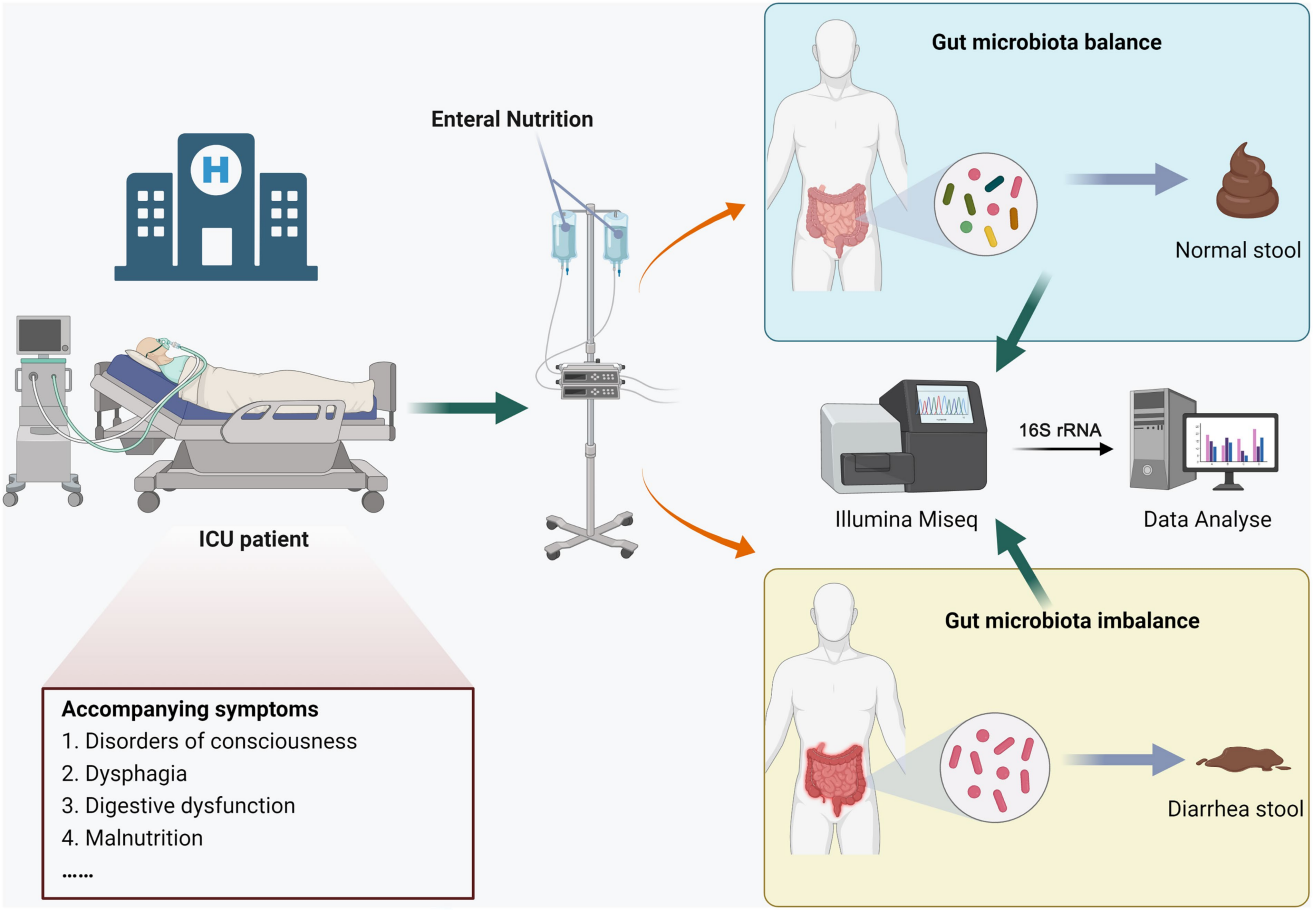
Experimental design and workflow.

This study was approved by the Ethics Review Committee of Henan Provincial People’s Hospital, and all participants signed informed consent forms. The process diagram was shown in Figure 1, hospitalized patients were divided into the END group (*n* = 7) and the no-END group (*n* = 7) according to whether diarrhea occurred during total enteral nutrition treatment in the ICU of our hospital in 2022. The inclusion criteria of the patients were as follows: (1) the type and dose of antibiotics were essentially the same; (2) there was no hypoalbuminemia; (3) there were no potassium preparations, antacids or prokinetic drugs; (4) probiotics or commensal bacteria were not used prior to collection of stool samples; and (5) there was no severe malnutrition, intestinal obstruction or digestive system diseases. At the same time, there were no statistically significant differences in age or sex between the two groups.

As the gold standard for stool collection, the rectal swab method was performed as previously described (Short et al., 2021). Briefly, the patient’s anus was first cleaned with soap, water and 70% alcohol. Then, a sterile swab was moistened with normal saline, inserted into the anus 4 ~ 5 cm, and rotated gently at the anal sphincter. A sample was taken from the anal crypt, and the swab with the patient’s stool was inserted into a sterile tube. Finally, the samples were frozen in liquid nitrogen and immediately stored at −80°C.

### DNA extraction and PCR amplification

Gut microbial DNA was extracted from no-END and END samples using the E.Z.N.A.® Soil DNA Kit (Omega Bio-Tek, Norcross, GA, U.S.) according to the manufacturer’s protocols. The final DNA concentration and purification were determined by a NanoDrop 2000 UV–vis spectrophotometer (Thermo Scientific, Wilmington, USA), and DNA quality was checked by 1% agarose gel electrophoresis.

The V3-V4 hypervariable regions of the bacterial 16S rRNA gene were amplified with primers 338F (5′- ACTCCTACGGGAGGCAGCAG-3′) and 806R (5’-GGACTACHVGGGTWTCTAAT-3′) by a thermocycler PCR system (GeneAmp 9,700, ABI, USA). The PCRs were conducted using the following program: 3 min of denaturation at 95°C; 27 cycles of 30 s at 95°C, 30 s for annealing at 55°C, and 45 s for elongation at 72°C; and a final extension at 72°C for 10 min. PCRs were performed in triplicate in a 20 μl mixture containing 4 μl of 5 × FastPfu Buffer, 2 μl of 2.5 mM dNTPs, 0.8 μl of each primer (5 μM), 0.4 μl of FastPfu Polymerase and 10 ng of template DNA. The resulting PCR products were extracted from a 2% agarose gel, further purified using the AxyPrep DNA Gel Extraction Kit (Axygen Biosciences, Union City, CA, USA) and quantified using QuantiFluor™-ST (Promega, USA) according to the manufacturer’s protocol.

### 16S rRNA gene sequence analysis

Before analysis, raw fastq sequence files were demultiplexed, quality-filtered by Trimmomatic and merged by FLASH with the following criteria: (i) the reads were truncated at any site receiving an average quality score b20 over a 50 bp sliding window; (ii) primers were exactly matched, allowing 2 nucleotide mismatching, and reads containing ambiguous bases were removed; and (iii) sequences whose overlap was longer than 10 bp were merged according to their overlap sequence.

The demultiplexed reads were clustered at 97% sequence identity into operational taxonomic units (OTUs) using UPARSE (version 10.0^1^), and chimeric sequences were identified and removed using UCHIME. The taxonomy of each 16S rRNA gene sequence was analyzed by the RDP Classifier algorithm (version 2.2^2^) in mothur^3^ against the Silva 132/16s_bacteria^4^ database using a confidence threshold of 70%. Sequencing was performed with an Illumina MiSeq PE300 platform.

### Statistical analysis

OTU taxonomies (from phylum to species) were determined based on NCBI. For the alpha-diversity analysis, the Ace, Chao index and Shannon and Simpson indices at the similarity level of 97% were calculated to compare the gut bacterial richness and diversity, respectively. For the beta-diversity analysis, analyses were visualized using the R package software. For example, a Venn diagram was implemented using the R package to show unique and shared OTUs. All statistical analyses were conducted using R package software. Differences between populations were analyzed using one-way ANOVA. Linear discriminant analysis (LDA) effect size (LEfSe (Segata et al., 2011)) was used to elucidate the biomarkers in the two groups. Those with an LDA score of 4.0 were considered to be important biomarkers in the no-END and END groups. The metagenomes were predicted from 16S data using Phylogenetic Investigation of Communities by Reconstruction of Unobserved States [PICRUSt (Langille et al., 2013)]. The functional genes were identified from the Kyoto Encyclopedia of Genes and Genomes (KEGG) database (Kanehisa and Goto, 2000). Specifically, PICRUSt is a software package for functional prediction based on 16S amplicon sequencing results. Firstly, the OTU abundance table of microbiota was standardized by PICRUSt (the PICRUSt process stores the KEGG Ortholog (KO) information corresponding to greengene id), that is, the effect of 16S marker gene copy number in the species genome was removed. Then through the greengene id corresponding to each OTU, the KO information and abundance corresponding to the OTU were obtained. Finally, according to the KEGG database, the descriptive information of each pathway was parsed, and the abundance of each functional category was calculated according to the OTU abundance. Furthermore, the G-TEST and Fisher test methods in STAMP software were used to test and visualize the significant differences in the predicted functional pathways by PICRUSt, and the *p* value threshold was 0.000.

## Results

### END alters the alpha diversity of the gut microbiota

To explore differences in the gut microbiota of healthy people and ICU patients with diarrhea, the Illumina MiSeq platform was used to perform high-throughput sequencing of the v3-v4 region of the bacterial 16S rRNA gene. In total, 593,989 high-quality sequences were obtained from 14 samples, with an average sequence length of 417 in the two groups. These sequences gathered 506,308 (420 types) OTUs with 97% sequence similarity, and each sample contained 31,105 to 40,088 OTUs.

**Figure 2:**
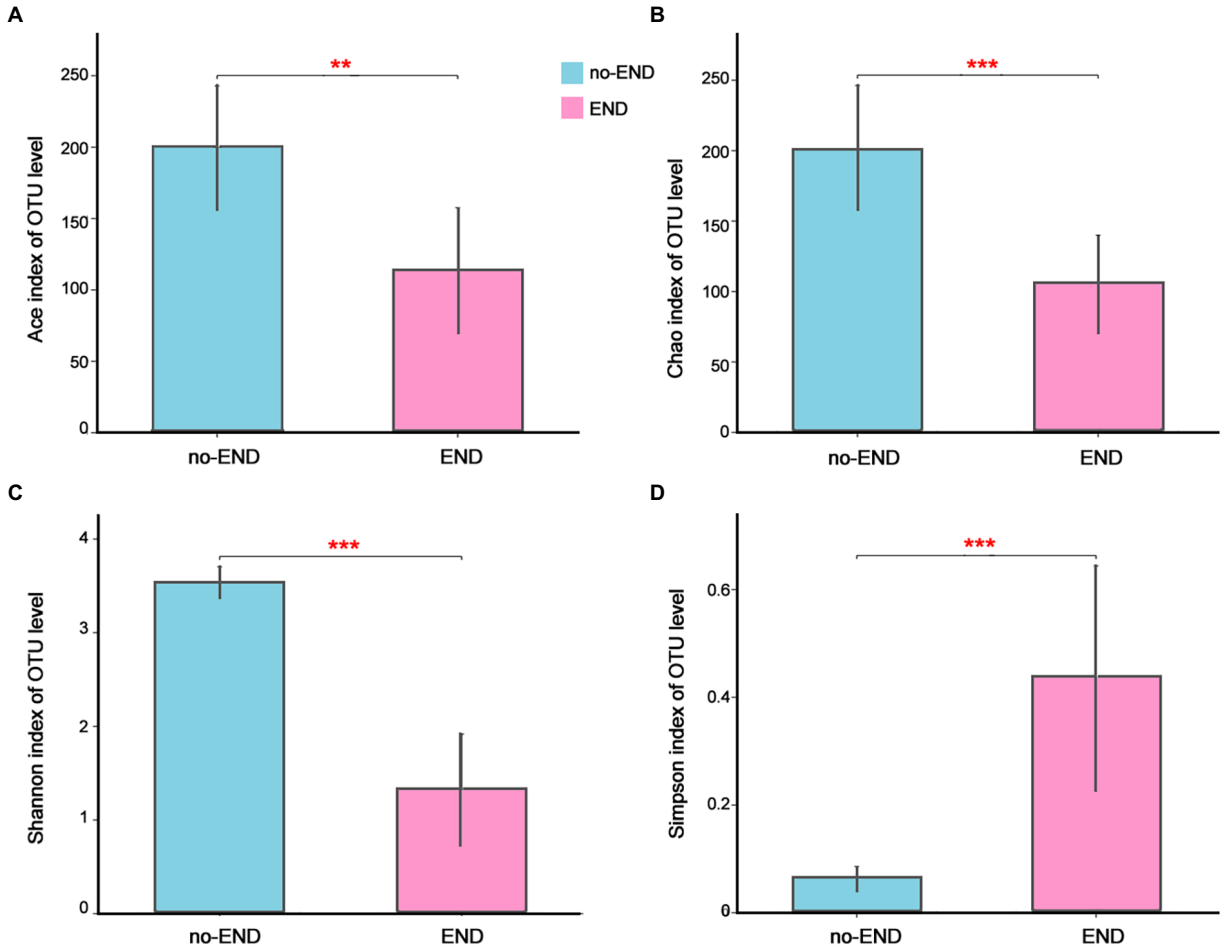
The richness and diversity of the gut microbiota in the two groups. **(A–D)** Ace, Chao, Shannon and Simpson indices of the OTU level, respectively. **p < 0.01 and ***p < 0.001. Data are presented as the means ± SD, Student’s *t* test (equal variance).

The Ace and Chao indices as well as Shannon and Simpson indices were used to explore the effect of END on the alpha-diversity of the gut microbiota in ICU patients. As shown in Figure 2, the Ace and Chao indices, which represent bacterial richness in the no-END and END groups, were 199.73 ± 42.78 vs. 113.92 ± 43.40 (*p* < 0.01; Figure 2A) and 200.67 ± 45.22 vs. 105.94 ± 33.73 (*p* < 0.001; Figure 2B), respectively. The bacterial diversity indices (Shannon and Simpson) in the no-END and END groups were 3.53 ± 0.16 vs. 1.33 ± 0.59 (*p* < 0.001; Figure 2C) and 0.06 ± 0.02 vs. 0.44 ± 0.20 (*p* < 0.001; Figure 1D), respectively. These results indicated that compared with no-END patients, the gut microbiota of END patients was disturbed, the richness and diversity of bacteria were all significantly reduced.

### Overall gut microbiota structures

**Figure 3:**
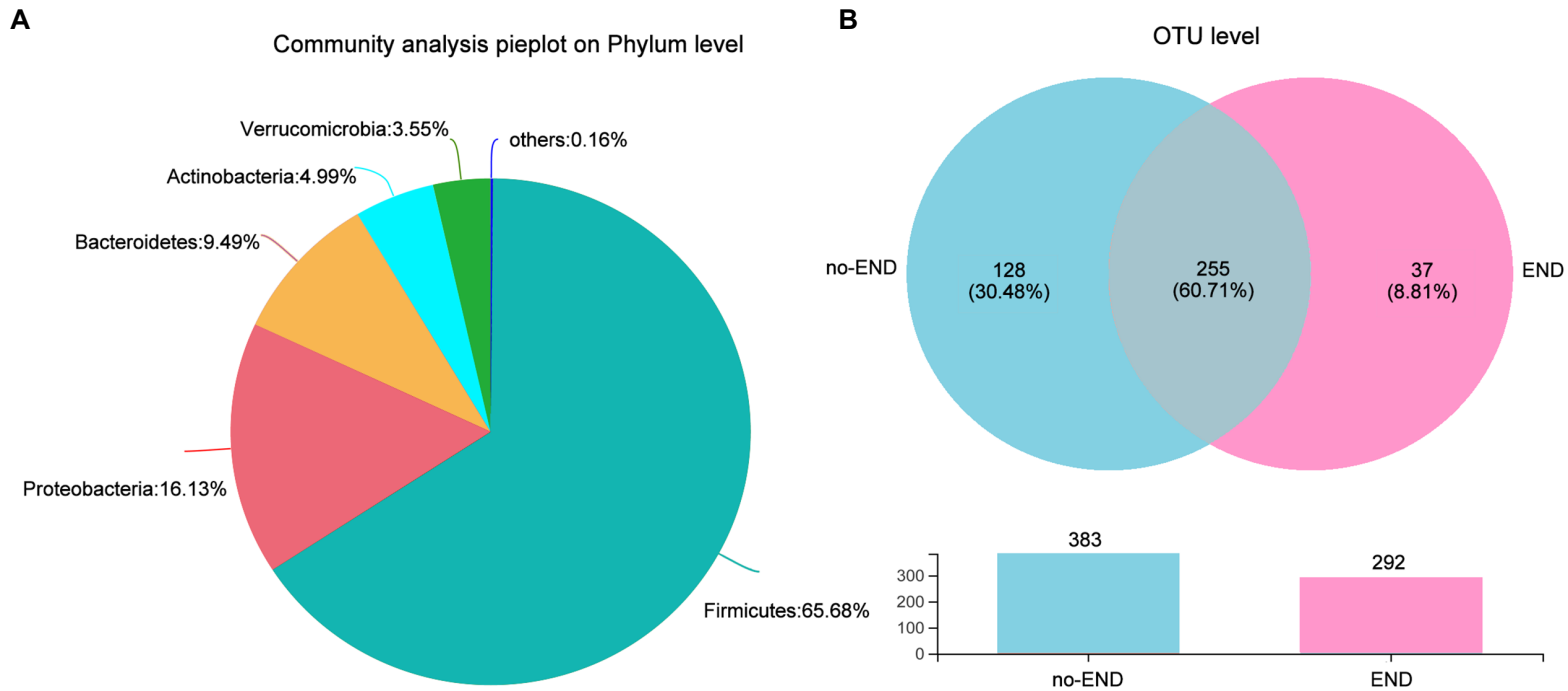
Characteristics of the overall microbiota composition in ICU patients receiving enteral nutrition. **(A)** The bacterial community in all samples at the phylum level. Less than 1% abundance of the phyla was merged into “others.” **(B)** Comparison of OTUs in the two groups by Venn diagram. The upper part is the two groups of unique or shared OTUs, and the lower part is the histogram of their total OTUs.

We then investigated the phylum-level bacterial composition of all ICU patient samples, regardless of whether diarrhea occurred. From 16S rRNA gene sequences, OTUs were identified as 13 prokaryotic phyla, and relative abundances of less than 1.00% were classified as “Others.” Specifically, the dominant bacterial phyla in all samples were *Firmicutes* (65.68%), *Proteobacteria* (16.13%), *Bacteroidetes* (9.49%), *Actinomycetes* (4.99%), *Verrucobacterium* (3.55%) and others (0.16%; Figure 3A).

To further study the shared and unique gut microbiota of the no-END and END groups, a Venn diagram was constructed to identify the types of OTUs in these two groups (Figure 3B). The results showed that there were 383 and 292 OTUs in the normal group and diarrhea group, respectively. While 225 OTUs (60.71%) were shared by both groups, 128 unique OTUs (30.48%) were present in the no-END group, and 37 unique OTUs (8.81%) newly emerged in the END group. These data strongly confirmed that END caused a significant reduction in gut microbial species, but there were still about 60% OTUs that were not affected by it, indicate the gut microbiota has robust intrinsic stability. However, the restructured host gut microbiota may be the underlying cause of END, and the answer possibly hidden in the remaining 40% of OTUs lost and gained by END patients.

### Comparing changes in the bacterial composition of END

**Figure 4:**
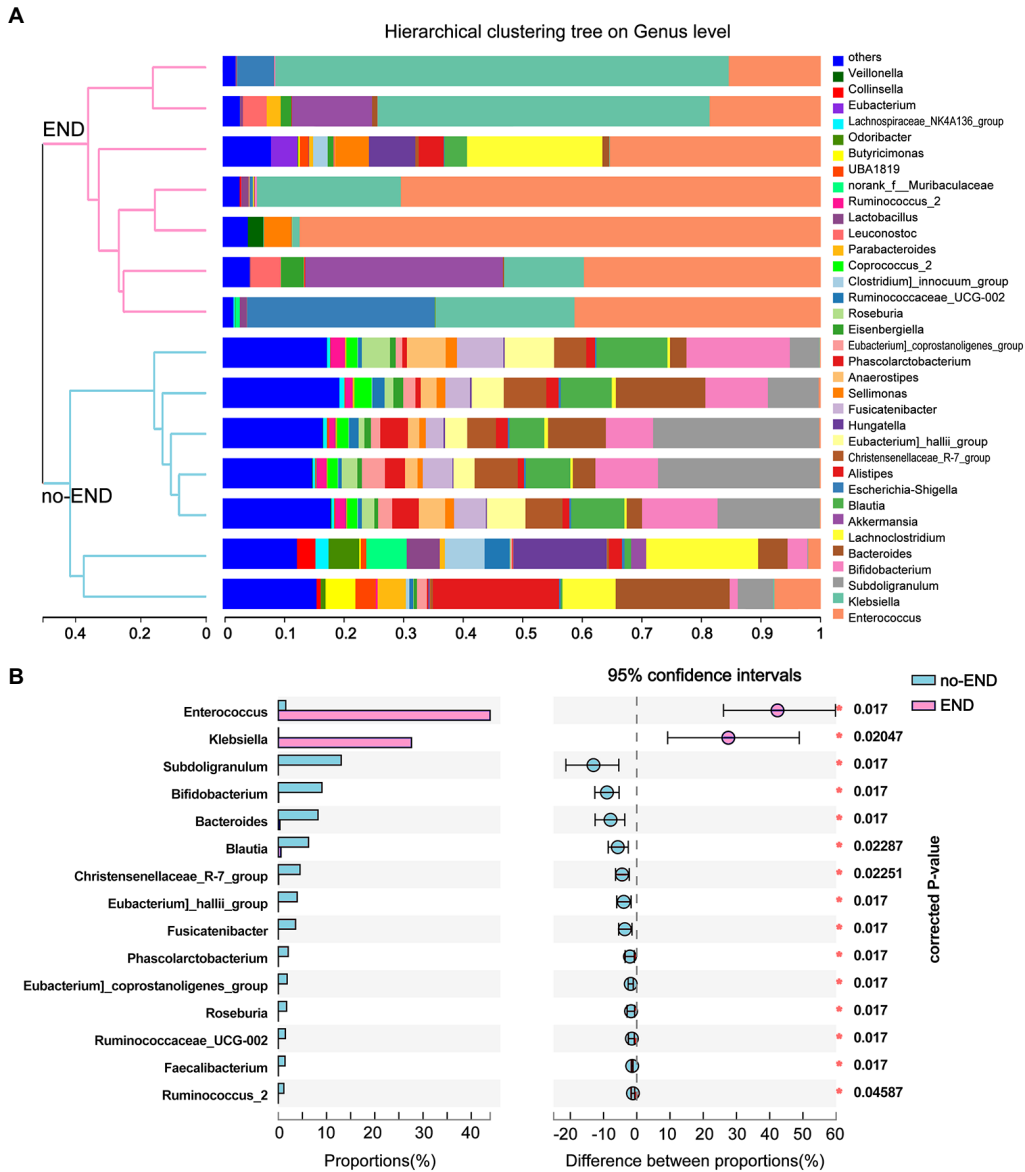
Flora distribution and LEfSe multilevel species difference. **(A)** Relative composition of genus-level microbiota in the two groups. The legend to the right of the graph indicates the color for each genus of bacterium represented. The low-abundance bacterial group included all bacterial classes with less than 2% total abundance. The hierarchical clustering tree on the left was calculated using the unweighted pair-group method with the arithmetic mean (UPGMA) method, and the relationship between samples was determined by Bray–Curtis distance and the average clustering method. **(B)** 16S rRNA profile comparisons of differentially abundant species between the no-END and END group samples using STAMP analysis at the genus level. The positive difference between proportions denotes a greater abundance in the END group (red), yet the negative difference between proportions shows a greater abundance in the no-END group (blue). The false discovery rate (FDR)-corrected *p* value was calculated based on a two-tailed Wilcoxon rank-sum test. Features with the top 15 (all corrected *p* < 0.05) were considered significant and were thus retained.

The microbial compositions of the no-END and END groups were compared at the genus level. As shown in Figure 4A, the most dominant gut microbiota in the two groups and their relative abundance ratios (Top10) were as follows. No-END group: *Subdoligranulum* (13.22%), *Bifidobacterium* (8.89%), *Blautia* (8.43%), *Bacteroides* (8.52%), *Blautia* (6.17%), *Alistipes* (4.69%), *Christensenellaceae_R-7_group*(4.48%), *Lachnoclostridium* (4.27%), *Eubacterium]_hallii_group* (3.85%) and *Fusicatenibacter* (3.52%); END group: *Enterococcus* (44.99%), *Klebsiella* (25.90%), other (6.93%), *Akkermansia* (6.71%), *Escherichia-Shigella* (5.02%), *Lachnoclostridium* (3.63%), *Sellimonas* (1.60%), *Leuconostoc* (1.26%), *Hungatella* (1.24%) and *Alistipes* (0.71%). The composition of dominant bacteria in the intestine clearly changed after diarrhea. In addition, the clustering tree using the Bray–Curtis distance algorithm on the left also clearly separated the two sets of samples.

By further comparing the differences in the genus-level gut microbiota between the two groups, we found 15 bacteria that were significantly altered (corrected *p* < 0.05). Specifically, compared with the no-END group, diarrhea led to the massive proliferation of *Enterococcus* and *Klebsiella*, while the other 13 microbiota represented by *Subdoligranulum*, *Bifidobacterium*, and *Blautia* were greatly reduced (Figure 4B).

### END causes gut microbiota disturbances

**Figure 5:**
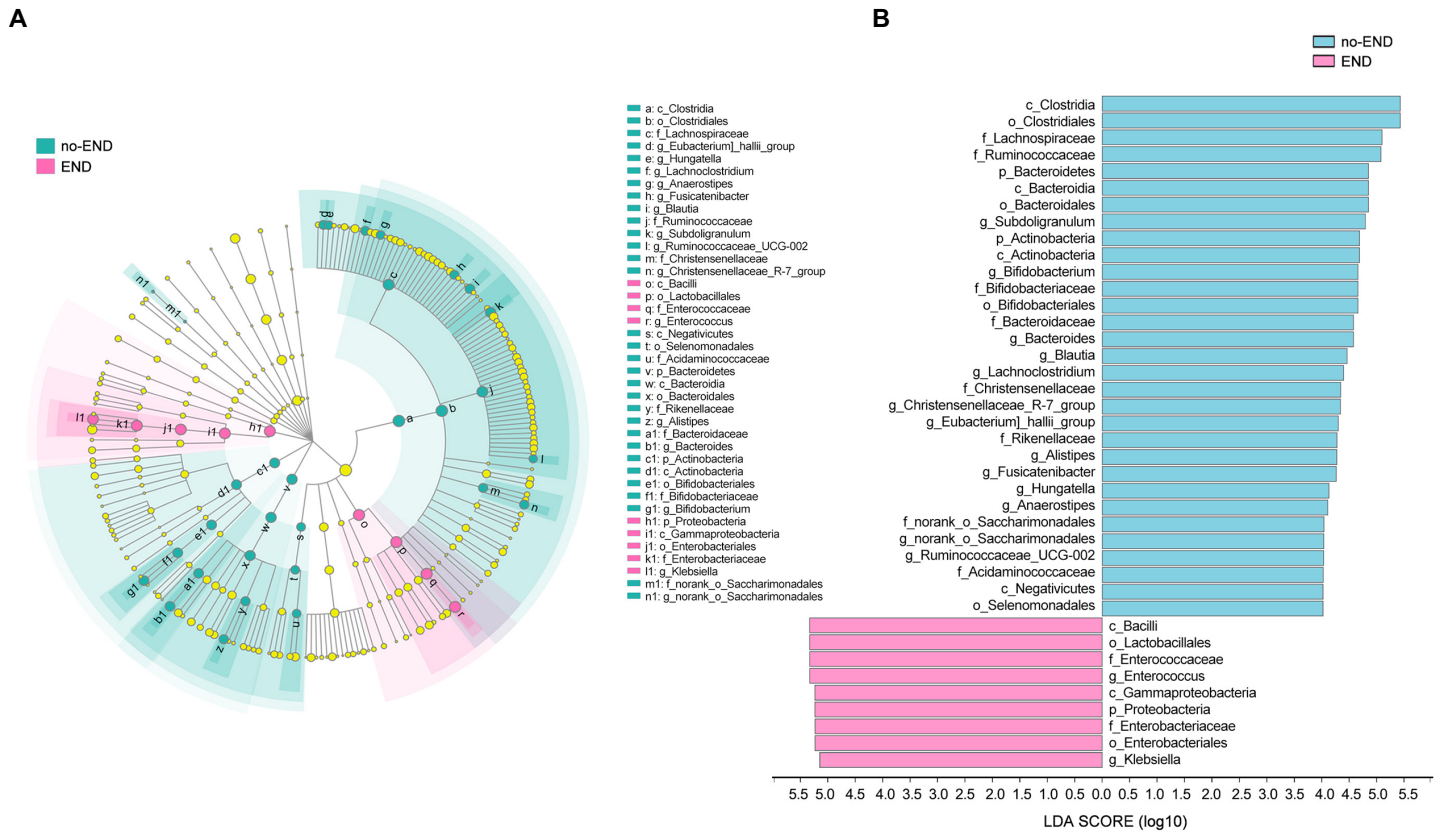
The specific altered taxa were identified by linear discriminant analysis (LDA) and effect size (LEfSe) analysis. **(A)** Phylogenetic tree in the cladogram of the specific differential taxa. Red and blue indicate increased or reduced abundance in the END group compared with the no-END group, respectively. **(B)** Histogram of taxa with LDA scores >4.0 and *p* values <0.05.

Diarrhea causes numerous changes in the gut microbiota of severely ill patients, leading to a state of ecological imbalance. To determine whether diarrhea affects the abundance of intestinal bacteria at various levels, LEfSe (LDA Score>4.0) was used to analyze the differences in microbial community composition between the two groups at the phylum, class, order, family, and genus levels to indicate the phylogenetic distribution. Compared with that of the no-END group, the gut microbiota of the END group was disordered (Figure 5A).

The results in Figure 5B clearly show 34 biomarkers in the no-END group and 11 biomarkers in the END group, regardless of their classification level. Specifically, the biomarkers (top 10) of the no-END group were o_*Clostridiales*, c_*Clostridia*, f_*Lachnospiraceae*, f_*Ruminococcaceae*, o_*Bacteroidales*, p_*Bacteroidetes*, c_*Bacteroidia*, g_*Subdoligranulum*, c_*Actinobacteria* and p_*Actinobacteria*, while c_*Bacilli*, o_*Lactobacillales*, g_*Enterococcus*, f_*Enterococcaceae*, c_*Gammaproteobacteria*, p_*Proteobacteria*, o_*Enterobacteriales*, f_*Enterobacteriaceae*, g_*Klebsiella* and k_*norank_d_Bacteria* were the biomarkers of the END group (‘p_’, phylum; ‘c_’, class; ‘f_’, family; ‘o_’, order; ‘g_’, genus).

### Differences in the gene function of the gut microbiota after END

To infer differentially abundant KEGG pathways associated with END based on the differences in bacterial abundance in the two groups, PICRUSt and STAMP were used to map microbial genes to KEGG databases.

**Figure 6:**
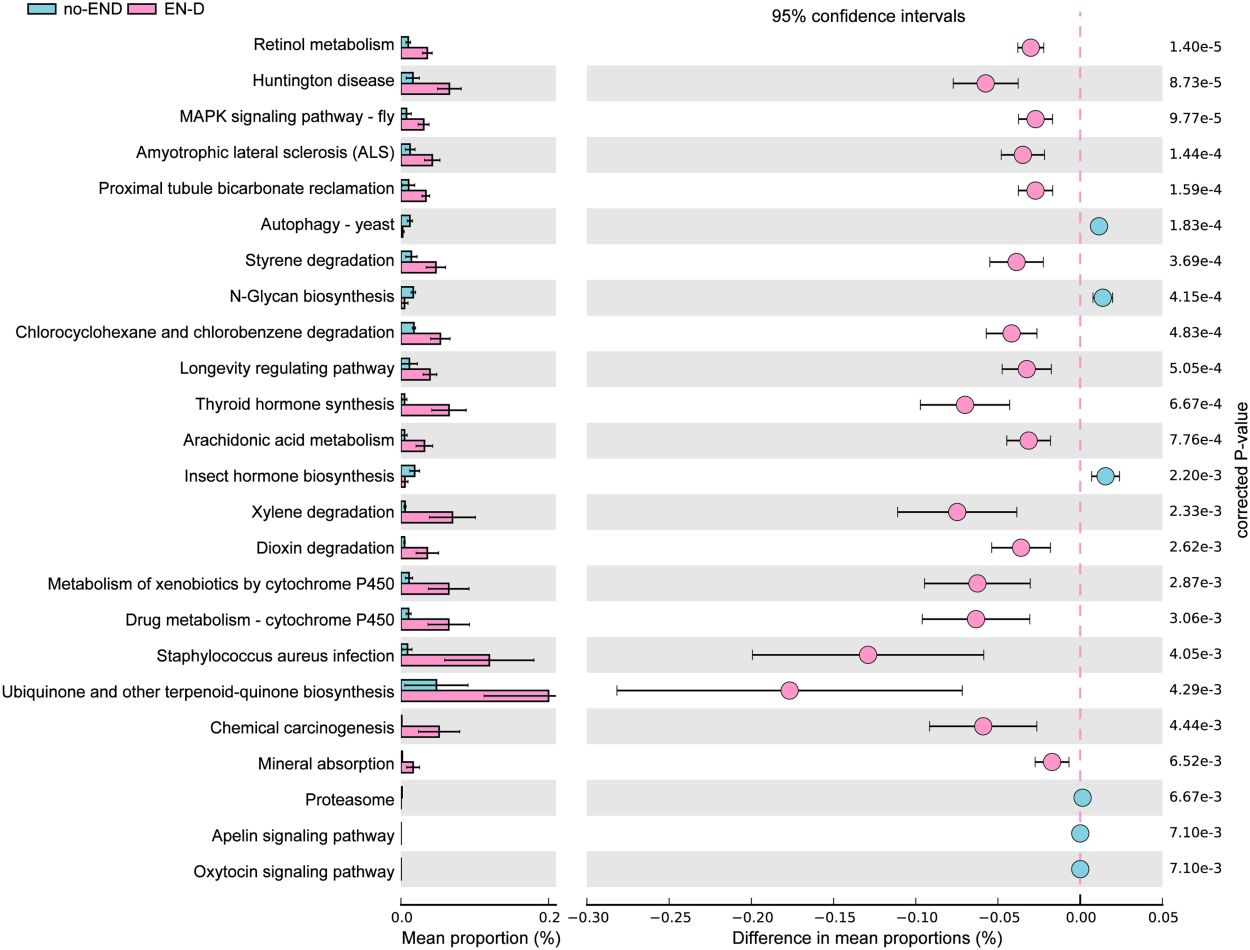
PICRUSt analysis of the KEGG pathways. The histogram on the left represents the abundance of KEGG Pathway level 3 as a percentage of all pathways in the two sets of samples, while the scatter diagram on the right is the corrected *p* value. STAMP using corrected *p* values was calculated based on two-tailed Welch’s inverted test. Features with corrected *p* < 0.05 and effect size >3 were considered significant and were thus retained.

The results in Figure 6 show that, due to the disorder of the gut microbiota in patients with diarrhea, the bacterial gene function was also significantly different than that in the no-END group. Specifically, by screening KEGG secondary pathways with *p* < 0.000, we identified 24 significantly different pathways. The 7 pathways that were abnormally activated after diarrhea were ascorbate and aldarate metabolism, retinol metabolism, aminobenzoate degradation, Huntington’s disease, tryptophan metabolism, the two-component system and the MAPK signaling pathway-fly. In addition, the other 17 pathways represented by Th17-cell differentiation, the IL-17 signaling pathway and necroptosis were significantly inactivated after END. Taken together, the predicted results of these biological functional pathways indicated that pathways mainly related to immunity and metabolism were significantly altered due to the imbalance of gut microbiota after diarrhea. Therefore, we speculate that this may be an important cause of END in ICU patients. However, it should be noted that these results are based on computational predictions, so subsequent experimental verification is essential.

## Discussion

The human gut microbiota contains 10^13^ to 10^14^ bacterial cells, which belong to hundreds of different species. The gut microbiota plays an important role in the physical and psychological processes of the human host (Sekirov et al., 2010; Simpson et al., 2021). Therefore, any dysbiosis can have a negative impact on health (Butel, 2014). In healthy individuals, the gut microbiota is mainly composed of bacteria that have a symbiotic or mutual relationship with the human host. Here, we describe an important result that links diarrhea with enteral nutritional support and gut microbiota. The results showed that ICU patients given enteral nutrition with diarrhea had significantly decreased gut microbial diversity compared with those without diarrhea. A reduction in microbial diversity disrupts intercellular integrity, leading to a leaky gut and increased gut permeability, which in turn leads to gut inflammation (Sharma and Tripathi, 2019). Furthermore, in this study, genus *Enterococcus* and *Klebsiella* were significantly increased when ICU patients presented with END. One study found that critically ill patients often develop intestinal dysbiosis, which is manifested by intestinal overgrowth and the emergence of opportunistic multidrug-resistant pathogens, such as genus *Enterococcus* (Zaborin et al., 2014; McDonald et al., 2016; Ojima et al., 2016). Another study found that *Enterococcus* are present in large numbers in the intestines of diarrheagenic *Escherichia coli* diarrhea patients, which are the main cause of diarrhea in children under 5 years of age worldwide (Gallardo et al., 2020). A North American study found that *Klebsiella* increased nearly 8-fold in small intestinal bacterial overgrowth (SIBO) subjects compared to non-SIBO populations (Leite et al., 2020). Moreover, a Spanish study characterized the intestinal microbiota in the stools of 57 patients with diarrhea from hospital and community-acquired *Clostridium difficile* infection and found that *Klebsiella* was one of the most abundant diarrhea-related bacteria in some patients (Hernandez et al., 2018). These results indicate that genus *Enterococcus* and *Klebsiella* may be pathogenic bacteria of various types of diarrhea, including END, and their excessive proliferation may play an important role in promoting the occurrence of diarrhea.

In contrast, we found that *Subdoligranulum*, *Bifidobacterium*, and *Bacteroides* were significantly reduced in the gut of END patients. One study in diarrhea-predominant irritable bowel syndrome (IBS) patients showed that fecal microbiota in IBS-D featured depleted *Subdoligranulum* (Liu et al., 2020). An animal study found that, compared with rotavirus-mediated newborn calves with diarrhea, the representative genera from *Subdoligranulum* and *Bacteroides* were closely related to healthy calves (Jang et al., 2019). *Bifidobacterium* can protect the intestinal epithelial barrier due to its anti-inflammatory regulatory function (Valdes et al., 2018). According to reports, supplementing *Bifidobacterium* in triple therapy can significantly improve the clearance of *Helicobacter pylori* in the gastrointestinal tract and reduce diarrhea (Sheu et al., 2002), reducing the duration of diarrhea in children with acute diarrhea (Yang et al., 2019) and reducing the symptoms of diarrhea in patients with IBS-D (Skrzydlo-Radomanska et al., 2020). Another study found that *Bifidobacterium infantis* reduces colonic permeability and inflammation, decreases interferon-gamma secretion, and maintains the balance of the intestinal system (Ewaschuk et al., 2008). Notably, *Bacteroidetes* are the most important anaerobic bacteria in the gut and are considered candidates for the next generation of probiotics due to their potential role in promoting host health (Wexler, 2007). This shows that *Bacteroides* are human symbiotic bacteria that can play positive roles in human health, and it is a promising therapeutic candidate. Interestingly, our data also showed that *Bacteroidetes* was one of the predominant bacteria in the no-EN samples, while a substantial decrease was observed after diarrhea. In a study of the primate Golden Snub-Nosed Monkey, the most obvious difference between healthy and diarrheal animals was that the *Bacteroides* abundance in diarrheal monkeys was reduced by nearly half (Zhu et al., 2018). These results demonstrated that diarrhea has a microbial component and that dysbiosis contributes to its occurrence. Consequently, the absence of *Subdoligranulum*, *Bifidobacterium*, and *Bacteroides* with this microbial structure could lead to increased susceptibility to END. On the other hand, *Bacteroides fragilis* ZY-312 has been reported to prevent AAD in rats (Zhang et al., 2018). These studies provide more evidence for the important role of *Bacteroidetes* in inhibiting the occurrence of diarrhea.

At present, the usual treatment for patients with diarrhea is probiotics (Li et al., 2021). Probiotics are non-pathogenic live microbial preparations that, when administered in adequate amounts, can alter the host’s gut microflora by improving its intestinal microbial balance, with beneficial effects (Hill et al., 2014). Different health conditions have proven to have beneficial effects with probiotics, such as lactose intolerance, hypercholesterolemia, traveler’s diarrhea, acute rotavirus diarrhea, radiotherapy-induced diarrhea, respiratory infections and others (Rondanelli et al., 2017). *Bifidobacterium* is currently one of the main species of probiotics. The probiotics represented by *Bifidobacterium* produce lactic acid, acetic acid, and propionic acid, which lower the intestinal pH and suppress the growth of various pathogenic bacteria, thereby reestablishing the balance of the gut flora. Interestingly, *Bifidobacterium* was also one of the main bacteria significantly reduced in END patients in our study. However, there is convincing evidence from multiple studies supporting the efficacy of probiotics in the treatment of multiple types of diarrhea (Capurso and Koch, 2021; Kambale et al., 2021; Zhang et al., 2021). However, in reality, the efficacy of probiotics for diarrhea in inpatients is not ideal (Allen et al., 2013). This may be either because the daily use of high-dose antibiotics in ICU patients with diarrhea offsets the effect of probiotics or because different types of diarrhea, such as END and AAD, lead to differences in patients’ sensitivity to current probiotics. It should also be noted that the effects of probiotics are strain-and dose-specific (Teitelbaum and Walker, 2002). Therefore, we aimed to clarify the type of diarrhea in ICU patients, adjust the types of probiotics, and carry out targeted supplementary interventions. For example, concomitant supplementation and increasing doses of *Bifidobacterium*, *Subdoligranulum* and *Bacteroides*, which were found to be significantly reduced after END in this study, may have unexpected ameliorating effects on diarrhea symptoms in ICU patients. Moreover, previous studies have shown that the gut microbiome has a close effect on diarrhea through metabolic pathways (Silverman et al., 2017; Rodriguez et al., 2020; Wei et al., 2020). Consistent with previous results, we also found that END mainly caused changes in metabolic pathways, such as inactivation of n-glycan biosynthesis and activation of retinol metabolism, arachidonic acid metabolism and drug metabolism - cytochrome P450 pathways. These results suggest that the gut microbiota may participate in the occurrence of END through these abnormally altered pathways.

Our study has several limitations. First, we analyzed a small number of samples; therefore, our results should be considered as an exploratory approach. However, our data provide valuable information about the importance of gut microbiota in understanding the mechanism of END diarrhea. Second, further longitudinal studies are needed to determine the causal relationship between the gut microbiome and END. Last, 16S rRNA sequencing technology itself also has some shortcomings, such as the inability to distinguish to the species level. In addition, PICRUSt analysis can only predict known functions of known microorganisms. The above shortcomings need to be further improved by metagenomic sequencing technology. Although these restrictions must be considered when interpreting our results, our data clearly demonstrate that enteral nutrition impairs the gut microbiota and exacerbates the diarrhea response. Thus, we highlight the necessity for timely protective microflora intervention for patients who already are or are about to undergo enteral nutrition treatment to avoid diarrhea.

In summary, these data highlight the comprehensive and detailed characteristics of changes in the gut microbiome at the time of diarrhea in ICU patients receiving EN treatment. By targeting inhibition of abnormal proliferation of *Enterococcus* and *Klebsiella*, and supplementing the loss of *Bifidobacterium*, *Subdoligranulum* and *Bacteroides* may be a novel and effective therapeutic strategy for clinical prevention and treatment of END.

## Data availability statement

The data presented in the study are deposited in the NCBI SRA repository, accession number: PRJNA885766, http://www.ncbi.nlm.nih.gov/bioproject/PRJNA885766.

## Ethics statement

The studies involving human participants were reviewed and approved by Ethics Committee of Henan Provincial People’s Hospital (2022-55). The patients/participants provided their written informed consent to participate in this study.

## Author contributions

SZ, LL, and XJ: conceptualization and supervision. WN, SZ, and LL: methodology. HZ, MJ, and SZ: investigation. WN and XJ: analysis, visualization, writing, review, and editing. All authors contributed to the article and approved the submitted version.

## Funding

This study was supported by grants from 23456 Talent Project of Henan Provincial People’s Hospital (No. ZC23456117) and Outstanding Innovative Talents Cultivation Funded Programs for Doctoral Students of Jinan University (No. 2022CXB015).

## Conflict of interest

The authors declare that the research was conducted in the absence of any commercial or financial relationships that could be construed as a potential conflict of interest.

## Publisher’s note

All claims expressed in this article are solely those of the authors and do not necessarily represent those of their affiliated organizations, or those of the publisher, the editors and the reviewers. Any product that may be evaluated in this article, or claim that may be made by its manufacturer, is not guaranteed or endorsed by the publisher.

## Footnotes

^1^ http://drive5.com/uparse/

^2^ http://sourceforge.net/projects/rdpclassifier/

^3^ https://www.mothur.org/

^4^ http://www.arb-silva.de

## References

[ref1] Adam S. Batson S. (1997). A study of problems associated with the delivery of enteral feed in critically ill patients in five ICUs in the UK. Intensive Care Med. 23, 261–266. doi: 10.1007/s001340050326, PMID: 9083227

[ref2] Allen S. J. Wareham K. Wang D. Bradley C. Hutchings H. Harris W.. (2013). Lactobacilli and bifidobacteria in the prevention of antibiotic-associated diarrhoea and Clostridium difficile diarrhoea in older inpatients (PLACIDE): a randomised, double-blind, placebo-controlled, multicentre trial. Lancet 382, 1249–1257. doi: 10.1016/S0140-6736(13)61218-0, PMID: 23932219

[ref3] Braun T. Di Segni A. BenShoshan M. Asaf R. Squires J. E. Farage Barhom S.. (2017). Fecal microbial characterization of hospitalized patients with suspected infectious diarrhea shows significant dysbiosis. Sci. Rep. 7:1088. doi: 10.1038/s41598-017-01217-1, PMID: 28439072 PMC5430810

[ref4] Butel M. J. (2014). Probiotics, gut microbiota and health. Med. Mal. Infect. 44, 1–8. doi: 10.1016/j.medmal.2013.10.002 24290962

[ref5] Capurso L. Koch M. (2021). Probiotics for the antibiotics associated diarrhea and for *Clostridium difficile* infection. Recenti Prog. Med. 112, 27–41. doi: 10.1701/3551.35255, PMID: 33576349

[ref6] De Lazzaro F. Alessandri F. Tarsitano M. G. Bilotta F. Pugliese F. (2022). Safety and efficacy of continuous or intermittent enteral nutrition in patients in the intensive care unit: systematic review of clinical evidence. JPEN J. Parenter. Enteral Nutr. 46, 486–498. doi: 10.1002/jpen.2316 34981842

[ref7] Ewaschuk J. B. Diaz H. Meddings L. Diederichs B. Dmytrash A. Backer J.. (2008). Secreted bioactive factors from *Bifidobacterium infantis* enhance epithelial cell barrier function. Am. J. Physiol. Gastrointest. Liver Physiol. 295, G1025–G1034. doi: 10.1152/ajpgi.90227.2008, PMID: 18787064

[ref8] Fan Y. Pedersen O. (2021). Gut microbiota in human metabolic health and disease. Nat. Rev. Microbiol. 19, 55–71. doi: 10.1038/s41579-020-0433-9 32887946

[ref9] Gallardo P. Izquierdo M. Vidal R. M. Soto F. Ossa J. C. Farfan M. J. (2020). Gut microbiota-metabolome changes in children with diarrhea by diarrheagenic *E. coli*. Front. Cell. Infect. Microbiol. 10:485. doi: 10.3389/fcimb.2020.00485, PMID: 33072619 PMC7531578

[ref10] Hernandez M. de Frutos M. Rodriguez-Lazaro D. Lopez-Urrutia L. Quijada N. M. Eiros J. M. (2018). Fecal microbiota of toxigenic *Clostridioides difficile*-associated diarrhea. Front. Microbiol. 9:3331. doi: 10.3389/fmicb.2018.03331, PMID: 30697203 PMC6341279

[ref11] Hill C. Guarner F. Reid G. Gibson G. R. Merenstein D. J. Pot B.. (2014). Expert consensus document. The international scientific association for probiotics and prebiotics consensus statement on the scope and appropriate use of the term probiotic. Nat. Rev. Gastroenterol. Hepatol. 11, 506–514. doi: 10.1038/nrgastro.2014.66, PMID: 24912386

[ref12] Hooda S. Minamoto Y. Suchodolski J. S. Swanson K. S. (2012). Current state of knowledge: the canine gastrointestinal microbiome. Anim. Health Res. Rev. 13, 78–88. doi: 10.1017/S1466252312000059, PMID: 22647637

[ref13] Jang J. Y. Kim S. Kwon M. S. Lee J. Yu D. H. Song R. H.. (2019). Rotavirus-mediated alteration of gut microbiota and its correlation with physiological characteristics in neonatal calves. J. Microbiol. 57, 113–121. doi: 10.1007/s12275-019-8549-1, PMID: 30456757 PMC7090552

[ref14] Kambale R. M. Nancy F. I. Ngaboyeka G. A. Kasengi J. B. Bindels L. B. Van der Linden D. (2021). Effects of probiotics and synbiotics on diarrhea in undernourished children: systematic review with meta-analysis. Clin. Nutr. 40, 3158–3169. doi: 10.1016/j.clnu.2020.12.026, PMID: 33446418

[ref15] Kanehisa M. Goto S. (2000). KEGG: Kyoto encyclopedia of genes and genomes. Nucleic Acids Res. 28, 27–30. doi: 10.1093/nar/28.1.27, PMID: 10592173 PMC102409

[ref16] Kang D. W. Adams J. B. Gregory A. C. Borody T. Chittick L. Fasano A.. (2017). Microbiota Transfer Therapy alters gut ecosystem and improves gastrointestinal and autism symptoms: an open-label study. Microbiome 5:10. doi: 10.1186/s40168-016-0225-7, PMID: 28122648 PMC5264285

[ref17] Krajmalnik-Brown R. Lozupone C. Kang D. W. Adams J. B. (2015). Gut bacteria in children with autism spectrum disorders: challenges and promise of studying how a complex community influences a complex disease. Microb. Ecol. Health Dis. 26:26914. doi: 10.3402/mehd.v26.26914, PMID: 25769266 PMC4359272

[ref18] Langille M. G. Zaneveld J. Caporaso J. G. McDonald D. Knights D. Reyes J. A.. (2013). Predictive functional profiling of microbial communities using 16S rRNA marker gene sequences. Nat. Biotechnol. 31, 814–821. doi: 10.1038/nbt.2676, PMID: 23975157 PMC3819121

[ref19] Leite G. Morales W. Weitsman S. Celly S. Parodi G. Mathur R.. (2020). The duodenal microbiome is altered in small intestinal bacterial overgrowth. PLoS One 15:e0234906. doi: 10.1371/journal.pone.0234906, PMID: 32645011 PMC7347122

[ref20] Li Y. Xia S. Jiang X. Feng C. Gong S. Ma J.. (2021). Gut microbiota and diarrhea: an updated review. Front. Cell. Infect. Microbiol. 11:625210. doi: 10.3389/fcimb.2021.625210, PMID: 33937093 PMC8082445

[ref21] Liu T. Gu X. Li L. X. Li M. Li B. Cui X.. (2020). Microbial and metabolomic profiles in correlation with depression and anxiety co-morbidities in diarrhoea-predominant IBS patients. BMC Microbiol. 20:168. doi: 10.1186/s12866-020-01841-4, PMID: 32552668 PMC7302156

[ref22] Lochs H. Allison S. P. Meier R. Pirlich M. Kondrup J. Schneider S.. (2006). Introductory to the ESPEN guidelines on enteral nutrition: terminology, definitions and general topics. Clin. Nutr. 25, 180–186. doi: 10.1016/j.clnu.2006.02.007, PMID: 16697086

[ref23] McDonald D. Ackermann G. Khailova L. Baird C. Heyland D. Kozar R.. (2016). Extreme dysbiosis of the microbiome in critical illness. mSphere. 1: e00199–16. doi: 10.1128/mSphere.00199-16 27602409 PMC5007431

[ref24] Ojima M. Motooka D. Shimizu K. Gotoh K. Shintani A. Yoshiya K.. (2016). Metagenomic analysis reveals dynamic changes of whole gut microbiota in the acute phase of intensive care unit patients. Dig. Dis. Sci. 61, 1628–1634. doi: 10.1007/s10620-015-4011-3, PMID: 26715502 PMC4875048

[ref25] Okonkwo A. Rimmer V. Walkden A. Brahma A. Carley F. McBain A. J.. (2020). Next-generation sequencing of the ocular surface microbiome: in health, contact lens wear, diabetes, trachoma, and dry eye. Eye Contact Lens 46, 254–261. doi: 10.1097/ICL.0000000000000697 32443013

[ref26] Pena-Gonzalez A. Soto-Giron M. J. Smith S. Sistrunk J. Montero L. Paez M.. (2019). Metagenomic signatures of gut infections caused by different Escherichia coli pathotypes. Appl. Environ. Microbio. 85:e01820–19. doi: 10.1128/AEM.01820-19, PMID: 31585992 PMC6881795

[ref27] Rodriguez C. Romero E. Garrido-Sanchez L. Alcain-Martinez G. Andrade R. J. Taminiau B.. (2020). Microbiota insights in clostridium Difficile infection and inflammatory bowel disease. Gut Microbes 12:1725220. doi: 10.1080/19490976.2020.1725220, PMID: 32129694 PMC7524151

[ref28] Rondanelli M. Faliva M. A. Perna S. Giacosa A. Peroni G. Castellazzi A. M. (2017). Using probiotics in clinical practice: where are we now? A review of existing meta-analyses. Gut Microbes 8, 521–543. doi: 10.1080/19490976.2017.1345414, PMID: 28640662 PMC5730384

[ref29] Schneider S. M. (2010). Microbiota and enteral nutrition. Gastroenterol. Clin. Biol. 34, S57–S61. doi: 10.1016/S0399-8320(10)70022-1 20889006

[ref30] Schneider S. M. Le Gall P. Girard-Pipau F. Piche T. Pompei A. Nano J. L.. (2000). Total artificial nutrition is associated with major changes in the fecal flora. Eur. J. Nutr. 39, 248–255. doi: 10.1007/s003940070003, PMID: 11395984

[ref31] Segata N. Izard J. Waldron L. Gevers D. Miropolsky L. Garrett W. S.. (2011). Metagenomic biomarker discovery and explanation. Genome Biol. 12:R60. doi: 10.1186/gb-2011-12-6-r60, PMID: 21702898 PMC3218848

[ref32] Sekirov I. Russell S. L. Antunes L. C. Finlay B. B. (2010). Gut microbiota in health and disease. Physiol. Rev. 90, 859–904. doi: 10.1152/physrev.00045.2009 20664075

[ref33] Sharma S. Tripathi P. (2019). Gut microbiome and type 2 diabetes: where we are and where to go? J. Nutr. Biochem. 63, 101–108. doi: 10.1016/j.jnutbio.2018.10.003, PMID: 30366260

[ref34] Sheu B. S. Wu J. J. Lo C. Y. Wu H. W. Chen J. H. Lin Y. S.. (2002). Impact of supplement with *Lactobacillus*-and *Bifidobacterium*-containing yogurt on triple therapy for *Helicobacter pylori* eradication. Aliment. Pharmacol. Ther. 16, 1669–1675. doi: 10.1046/j.1365-2036.2002.01335.x, PMID: 12197847

[ref35] Short M. I. Hudson R. Besasie B. D. Reveles K. R. Shah D. P. Nicholson S.. (2021). Comparison of rectal swab, glove tip, and participant-collected stool techniques for gut microbiome sampling. BMC Microbiol. 21:26. doi: 10.1186/s12866-020-02080-3, PMID: 33446094 PMC7809826

[ref36] Silverman M. A. Konnikova L. Gerber J. S. (2017). Impact of antibiotics on necrotizing *Enterocolitis* and antibiotic-associated diarrhea. Gastroenterol. Clin. N. Am. 46, 61–76. doi: 10.1016/j.gtc.2016.09.010, PMID: 28164853 PMC5314436

[ref37] Simpson C. A. Diaz-Arteche C. Eliby D. Schwartz O. S. Simmons J. G. Cowan C. S. M. (2021). The gut microbiota in anxiety and depression - a systematic review. Clin. Psychol. Rev. 83:101943. doi: 10.1016/j.cpr.2020.101943, PMID: 33271426

[ref38] Singer P. (2019). Preserving the quality of life: nutrition in the ICU. Crit. Care 23:139. doi: 10.1186/s13054-019-2415-8, PMID: 31200741 PMC6570623

[ref39] Singer P. Blaser A. R. Berger M. M. Alhazzani W. Calder P. C. Casaer M. P.. (2019). ESPEN guideline on clinical nutrition in the intensive care unit. Clin. Nutr. 38, 48–79. doi: 10.1016/j.clnu.2018.08.037, PMID: 30348463

[ref40] Skrzydlo-Radomanska B. Prozorow-Krol B. Cichoz-Lach H. Majsiak E. Bierla J. B. Kosikowski W.. (2020). The effectiveness of synbiotic preparation containing Lactobacillus and Bifidobacterium probiotic strains and short chain fructooligosaccharides in patients with diarrhea predominant irritable bowel syndrome-a randomized double-blind, placebo-controlled study. Nutrients 12:1999. doi: 10.3390/nu12071999, PMID: 32635661 PMC7400954

[ref41] Teitelbaum J. E. Walker W. A. (2002). Nutritional impact of pre-and probiotics as protective gastrointestinal organisms. Annu. Rev. Nutr. 22, 107–138. doi: 10.1146/annurev.nutr.22.110901.145412 12055340

[ref42] Theilla M. Rattanachaiwong S. Kagan I. Rigler M. Bendavid I. Singer P. (2021). Validation of GLIM malnutrition criteria for diagnosis of malnutrition in ICU patients: an observational study. Clin. Nutr. 40, 3578–3584. doi: 10.1016/j.clnu.2020.12.021, PMID: 33413910

[ref43] Valdes A. M. Walter J. Segal E. Spector T. D. (2018). Role of the gut microbiota in nutrition and health. BMJ 361:k2179. doi: 10.1136/bmj.k2179, PMID: 29899036 PMC6000740

[ref44] Wei W. Wang H. F. Zhang Y. Zhang Y. L. Niu B. Y. Yao S. K. (2020). Altered metabolism of bile acids correlates with clinical parameters and the gut microbiota in patients with diarrhea-predominant irritable bowel syndrome. World J. Gastroenterol. 26, 7153–7172. doi: 10.3748/wjg.v26.i45.7153, PMID: 33362374 PMC7723672

[ref45] Wexler H. M. (2007). Bacteroides: the good, the bad, and the nitty-gritty. Clin. Microbiol. Rev. 20, 593–621. doi: 10.1128/CMR.00008-07, PMID: 17934076 PMC2176045

[ref46] Whelan K. (2007). Enteral-tube-feeding diarrhoea: manipulating the colonic microbiota with probiotics and prebiotics. Proc. Nutr. Soc. 66, 299–306. doi: 10.1017/S0029665107005551, PMID: 17637081

[ref47] Whelan K. Judd P. A. Tuohy K. M. Gibson G. R. Preedy V. R. Taylor M. A. (2009). Fecal microbiota in patients receiving enteral feeding are highly variable and may be altered in those who develop diarrhea. Am. J. Clin. Nutr. 89, 240–247. doi: 10.3945/ajcn.2008.26219, PMID: 19056551

[ref48] Wiesen P. Van Gossum A. Preiser J. C. (2006). Diarrhoea in the critically ill. Curr. Opin. Crit. Care 12, 149–154. doi: 10.1097/01.ccx.0000216583.64804.46 16543792

[ref49] Wischmeyer P. E. (2021). Overcoming challenges to enteral nutrition delivery in critical care. Curr. Opin. Crit. Care 27, 169–176. doi: 10.1097/MCC.0000000000000801, PMID: 33395086 PMC8276753

[ref50] Xie Y. Tian R. Wang T. Jin W. Hou Y. Zhou Z.. (2021). A prediction model of enteral nutrition complicated with severe diarrhea in ICU patients based on CD55. Ann. Palliat. Med. 10, 1610–1619. doi: 10.21037/apm-20-1050, PMID: 33222452

[ref51] Yang B. Lu P. Li M. X. Cai X. L. Xiong W. Y. Hou H. J.. (2019). A meta-analysis of the effects of probiotics and synbiotics in children with acute diarrhea. Medicine 98:e16618. doi: 10.1097/MD.0000000000016618, PMID: 31517810 PMC6750275

[ref52] Zaborin A. Smith D. Garfield K. Quensen J. Shakhsheer B. Kade M.. (2014). Membership and behavior of ultra-low-diversity pathogen communities present in the gut of humans during prolonged critical illness. MBio 5, e01361–e01314. doi: 10.1128/mBio.01361-14 25249279 PMC4173762

[ref53] Zhang W. Zhu B. Xu J. Liu Y. Qiu E. Li Z.. (2018). Bacteroides fragilis protects against antibiotic-associated diarrhea in rats by modulating intestinal defenses. Front. Immunol. 9:1040. doi: 10.3389/fimmu.2018.01040, PMID: 29868005 PMC5954023

[ref54] Zhang X. L. Chen M. H. Geng S. T. Yu J. Kuang Y. Q. Luo H. Y.. (2021). Effects of probiotics on diarrhea and CD4 cell count in people living with HIV: a systematic review and meta-analysis. Front. Pharmacol. 12:570520. doi: 10.3389/fphar.2021.570520, PMID: 34349637 PMC8326399

[ref55] Zhao R. Wang Y. Huang Y. Cui Y. Xia L. Rao Z.. (2017). Effects of fiber and probiotics on diarrhea associated with enteral nutrition in gastric cancer patients: a prospective randomized and controlled trial. Medicine 96:e8418. doi: 10.1097/MD.0000000000008418, PMID: 29069041 PMC5671874

[ref56] Zhou Y. Zhu X. Hou H. Lu Y. Yu J. Mao L.. (2018). Characteristics of diarrheagenic *Escherichia coli* among children under 5 years of age with acute diarrhea: a hospital based study. BMC Infect. Dis. 18:63. doi: 10.1186/s12879-017-2936-1, PMID: 29390982 PMC5796495

[ref57] Zhu H. Zeng D. Wang Q. Wang N. Zeng B. Niu L.. (2018). Diarrhea-associated intestinal microbiota in captive Sichuan Golden snub-nosed monkeys (*Rhinopithecus roxellana*). Microbes Environ. 33, 249–256. doi: 10.1264/jsme2.ME17163, PMID: 30047510 PMC6167115

